# Apolipoprotein J/Clusterin in Human Erythrocytes Is Involved in the Molecular Process of Defected Material Disposal during Vesiculation

**DOI:** 10.1371/journal.pone.0026033

**Published:** 2011-10-07

**Authors:** Marianna H. Antonelou, Anastasios G. Kriebardis, Konstantinos E. Stamoulis, Ioannis P. Trougakos, Issidora S. Papassideri

**Affiliations:** 1 Department of Cell Biology and Biophysics, Faculty of Biology, University of Athens, Panepistimiopolis, Athens, Greece; 2 Department of Medical Laboratories, Faculty of Health and Caring Professions, Technological and Educational Institute of Athens, Athens, Greece; 3 Blood Transfusion Center, Nikea, Piraeus, Greece; Faculdade de Medicina, Universidade de São Paulo, Brazil

## Abstract

**Background:**

We have showed that secretory Apolipoprotein J/Clusterin (sCLU) is down-regulated in senescent, stressed or diseased red blood cells (RBCs). It was hypothesized that sCLU loss relates to RBCs vesiculation, a mechanism that removes erythrocyte membrane patches containing defective or potentially harmful components.

**Methodology/Principal Findings:**

To investigate this issue we employed a combination of biochemical and microscopical approaches in freshly prepared RBCs or RBCs stored under standard blood bank conditions, an *in vitro* model system of cellular aging. We found that sCLU is effectively exocytosed *in vivo* during membrane vesiculation of freshly prepared RBCs. In support, the RBCs' sCLU content was progressively reduced during RBCs *ex vivo* maturation and senescence under cold storage due to its selective exocytosis in membrane vesicles. A range of typical vesicular components, also involved in RBCs senescence, like Band 3, CD59, hemoglobin and carbonylated membrane proteins were found to physically interact with sCLU.

**Conclusions/Significance:**

The maturation of RBCs is associated with a progressive loss of sCLU. We propose that sCLU is functionally involved in the disposal of oxidized/defected material through RBCs vesiculation. This process most probably takes place through sCLU interaction with RBCs membrane proteins that are implicit vesicular components. Therefore, sCLU represents a pro-survival factor acting for the postponement of the untimely clearance of RBCs.

## Introduction

Secretory Apolipoprotein J/Clusterin (sCLU) is a heterodimeric glycoprotein that is found in all human fluids and tissues having a ubiquitous expression pattern. sCLU exhibits an impressive repertoire of binding partners [1]. It is characterized by an extremely sensitive responsiveness to external stress stimuli [2]. sCLU reported physiological functions include complement inhibition, clearance of cellular debris [3], chaperoning of denatured proteins [4] and regulation of cell death [5].

The red blood cells (RBCs) aging process is a tightly regulated and time-dependent, but not necessarily linear sum of molecular events that leads to the non-random removal of senescent cells from the circulation [6]. During aging RBCs lose water, energy, proteins, membrane area and deformability. Membrane microvesiculation is an integral part of the RBCs maturation that is accelerated in older cells [7]. It is a process that challenges RBCs by irreversible surface area and hemoglobin (Hb) loss. However, it may also function in favour of the mature cells through the scavenging of damaged, non-functional or signalling-effective cell components that are released with the vesicles, preventing thus the premature death of RBCs [8]. Despite the critical effect(s) of RBCs aging in hematological and non-hematological diseases as well as in transfusion medicine, many issues of the RBCs maturation and senescence remain elusive.

Although RBCs storage in the cold, under blood banking conditions, is far from being considered analogous to the physiologic aging process *in vivo*, it has been widely used as a model system for studying human RBCs aging, as stored RBCs progressively express most of the molecular signals of *in vivo* aging and erythrophagocytosis [9]. In fact, almost the whole set of structural and functional deteriorations of stored RBCs that collectively referred to as “RBCs storage lesion”, exhibits impressive resemblance to RBCs aging *in vivo* [10]. The main biophysical effect of storage is the progressively increased vesiculation and the subsequent changes in RBCs mechanical and rheological properties. The oxidative mechanisms that drive normal RBCs senescence [11], [12] are probably related to the storage lesions through their effect on the physiological aging and vesiculation under storage [13], [14]. Supportively, RBCs that have been stored under the antioxidant and membrane-stabilizing effect of mannitol exhibit a milder expression pattern of senescence marks and vesiculation compared to those stored in autologous plasma (Au-Pl) [15].

We have found (Antonelou et al., accompanying paper) that the RBCs membrane-associated sCLU decreases during organismal aging, following organism exposure to acute stress, in patients with congenital hemolytic anemia, as well as during RBCs *in vivo* senescence. To elucidate the biological role of sCLU in mature RBCs we monitored sCLU levels and binding profile in erythrocytes during their *ex vivo* aging in blood bank conditions. We found that sCLU is progressively depleted from the stored RBCs and is accumulated in the oxidized vesicles released from them, probably through its physical interactions with an array of vesicles-associated proteins. Our novel findings clearly imply a functional role for sCLU in the physiology of the human RBCs and in the molecular processes of aging and vesiculation.

## Materials and Methods

### Ethics

The study has been submitted and has been approved by the Research Bioethics and BioSecure Committee of the Faculty of Biology/University of Athens. Investigations were carried out in accordance with the principles of the Declaration of Helsinki. Written informed consent was obtained from all blood donors participating in this study.

### Subjects and RBCs storage in blood bank conditions

Venous blood of 22 healthy, adult, young and non-smoking volunteers was used in the present study. Nine of them donated a small volume of peripheral blood that was used in the immunoprecipitation and confocal laser scanning microscopy (CLSM) experiments, shortly after sampling. Eligible blood donors (N = 13) donated larger blood volume (450±50 ml) and packed RBCs were afterwards stored in anticoagulant, preservative and/or additive solutions for 5–6 weeks at 4°C, according to the standard blood banking protocols. Collection and processing of blood for cold storage, as well as isolation, storage and sampling of packed RBCs under various conditions were performed as previously described [15]. Briefly, blood from seven donors was collected in citrate-phosphate-dextrose-adenine (CPDA) double-pack container systems. Packed RBCs were isolated and stored in autologous plasma (Au-Pl) at 4°C. Blood from six different donors was also collected in a quadruple citrate-phosphate-dextrose (CPD)–saline-adenine-glucose-mannitol (SAGM) top-and-bottom bag system and anti-coagulated with 70 ml of CPD (26.30 g/L sodium citrate dihydrate, 3.27 g/L citric acid monohydrate, 2.51 g/L sodium phosphate dihydrate, 25.00 g/L glucose monohydrate, pH 5.6). RBCs concentrates were also produced. SAGM was then added to the units after leukodepletion by in-line filtration. Au-Pl or CPD-SAGM units were stored at 4°C for the whole storage period (35 or 42 days, respectively). For the storage analyses, samples were withdrawn during the first 3–4 days of storage and weekly thereafter. Four units of the Au-Pl-stored RBCs were used for the collection of vesicles and sampled on the first day of storage and afterwards on days 11, 17, 20, 27 and 34. As controls, RBCs and vesicles of days 0–4 of the packed RBCs units were used.

### Erythrocytes and vesicles isolation - preparation of membrane and cytosol fractions

Purified RBCs were isolated and fractionated in membrane and cytosol preparations as described (Antonelou et al., accompanying paper). Vesicles were isolated from the plasma of stored RBCs concentrates by ultracentrifugation as detailed elsewhere [16]. Protein concentration was determined using the Bradford protein assay. For the in vitro oxidation experiments 30 ml of packed stored purified RBCs (CPD-SAGM leukodepleted units, day 42 of storage) per sample were diluted in phosphate buffered saline (PBS) containing 2.5 mM t-butyl hydroperoxide (tBHP). Following incubation for 30 min at 37°C the cells pelleted and the supernatant was centrifuged to collect the RBC-derived membrane vesicles.

### Immunoblotting and immunoprecipitation analyses

Equal amounts (12–200 µg) of RBCs fractions or vesicles protein were loaded in Laemmli gels, blotted to nitrocellulose membranes and probed with antibodies as previously described [15]. Immunoblots were developed using chemiluminescence (ECL) and quantified by scanning densitometry (Gel Analyzer v.1.0 image-processing program). For immunoprecipitation experiments, 1 mg of isolated RBCs membrane proteins were resuspended in Nonidet P-40 (NP-40) containing lysis buffer (50 mM Tris-HCl pH 8.0, 150 mM NaCl, 1% NP-40, 0.6 mM phenylmethylsulfonyl fluoride and proteinase inhibitor cocktail) as previously described [17]. Supernatants were incubated with the specific primary antibodies, for two hours at 4°C. Following the addition of Protein-G Sepharose, the precipitates were analyzed by immunoblotting. For the detection of carbonylated components into the immunoprecipitated material, the sCLU- and band 3-related immunoprecipitates were derivatized by 2,4-dinitrophenylhydrazine solution (at 1∶4 v/v ratio) for 30 min at room temperature before neutralization of the mixture with 1.5 vol of neutralization solution (Oxyblot® detection kit).

### Confocal Laser Scanning Microscope (CLSM) immunofluorescence co-immunolocalization and Transmission Electron microscopy (TEM) immunogold localization

For double immunofluorescence staining, RBCs were fixed with 90% methanol in PBS, permeabilized in PBS containing 0.05% Triton X-100 and blocked with 3% bovine serum albumin and 0.1% Tween-20 in PBS [18]. Cells were probed with the appropriate primary and secondary antibodies conjugated to fluorescein isothiocyanate (FITC) or rhodamine. Slides were observed under a Digital Eclipse C1 (Nikon, Melville, NY) CLSM and recorded at the same exposure time. Controls were prepared as described previously [15] and were free of immunoreactivity (data not shown). sCLU localization in RBCs or vesicles by immunogold TEM was done as previously described (Antonelou et al., accompanying paper). Thin sections were probed with the sCLU antibody and immunoglobulins G (IgGs) conjugated to 15 nm gold particles and examined on a Phillips EM 300 electron microscope operating at 80 kV accelerating voltage.

### Statistical analysis

Presented experiments have been analyzed for statistical significance using the one-way analysis of variance (ANOVA), the independent t-test or the chi-squared test as previously described (Antonelou et al., accompanying paper); significance was accepted at p<0.05. Individual protein levels were quantified against a reference membrane protein further normalized to the corresponding controls (samples stored for a short period). All experiments shown have been repeated at least two times unless otherwise stated.

### Material Supplies

The monoclonal antibodies against Band 3 (B9277) and actin (A5316), the horseradish peroxidise (HRP)-conjugated secondary antibodies (A-5420), as well as the Protease Inhibitor Cocktail, tBHP, α-cellulose, microcrystalline cellulose (Sigmacell type 50) and common chemicals and buffers were obtained from Sigma-Aldrich (Germany). Polyclonal antibodies against Hb (GR800GAP) and peroxiredoxin-2 (SP5464) were obtained from Europa Bioproducts (Cambridge) and from Acris GmbH (Hiddenhausen, Germany), respectively. Primary antibodies against sCLU (sc-6419) and Band 3 (sc-20657) as well as secondary antibodies conjugated to fluorescein isothiocyanate or rhodamine were from Santa Cruz Biotechnology (Santa Cruz, CA). The polyclonal antibody against CD59 was obtained from R&D Systems (AF 1987). HRP-conjugated secondary antibodies (NA 934) and enhanced chemiluminescence Western blot detection kit were from GE Healthcare Amersham (Piscataway, NJ). HRP-conjugated secondary antibodies (P0161) were from DakoCytomation (Glostrup, Denmark). The polyclonal antibody against dinitrophenylhydrazone (DNP) residues was obtained from Millipore (Oxyblot® detection kit, S7150, Chemicon, Temecula, CA). Unicryl acrylic resin and IgGs conjugated to 15 nm gold particles were obtained from British Biocell International (Cardiff, Wales, UK). Bradford protein assay was from Bio-Rad (Hercules, CA). Quadruple CPD–SAGM top-and-bottom bag systems were obtained from Baxter (Rome, Italy). Gel Analyzer v.1.0 image-processing system and software was obtained from Biosure (Athens, Greece). The monoclonal antibody against stomatin and the antiserum against protein 4.1R were kindly provided by Prof. R. Prohaska (Department of Medical Biochemistry, Medical University of Vienna, Austria) and Prof. J. Delaunay (Service d' Hématologie, Hôpital de Bicetre, Le Kremlin-Bicetre, France) respectively.

## Results

### sCLU exocytosis during *in vivo* RBCs membrane vesiculation

**Figure 1:**
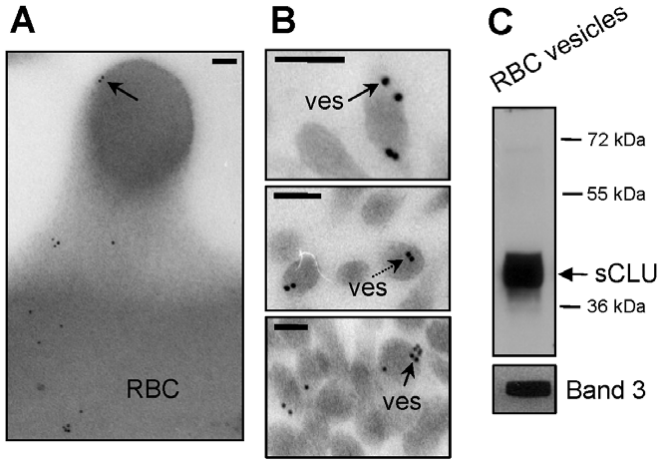
sCLU exocytosis to RBCs-derived vesicles. (A, B) TEM immunogold localization of sCLU in RBCs membrane protrusions (A) and vesicles (ves) (B) collected from fresh units of stored RBCs (N = 2, young healthy donors). Solid or dashed arrows indicate sCLU immunogold localization at the periphery or the cytosol of the vesicles, respectively. (C) Representative immunoblot analysis of RBCs-derived purified vesicles (N = 2) probed with either polyclonal anti-sCLU or with monoclonal anti-Band 3 antibodies. Molecular weight markers are indicated to the right of the blot. Bars in (A), (B), 100 nm.

The continuous release of Hb-containing membrane vesicles has been described as an integral part of the erythrocyte maturation and senescence *in vivo* [7]. Since vesiculation is accompanied by membrane surface and protein loss, we queried whether the recently described selective loss of sCLU from the membrane of senescent or stressed RBCs (Antonelou et al., accompanying paper) was associated with its exocytosis, as a component of the released membrane vesicles. Considering the low concentration and the technical difficulties in obtaining pure, RBCs-derived vesicles from human plasma, for vesicles' isolation purposes we used the plasma of fresh (namely a few hours after blood donation), leukodepleted packed RBCs units, from young, healthy donors (N = 2), collected in blood bank conditions (SAGM). Immunogold localization analysis of sCLU in those RBCs (N = 2) demonstrated the presence of sCLU not only in the membrane and the cytosol but also at the protrusions of budding RBCs membrane areas (Fig. 1A). The same analysis in ultrathin sections of RBC-derived vesicles (N = 2) revealed sCLU-specific immunogold particles primarily at the periphery (Fig. 1B; arrows), yet at the cytosol of the vesicles too (Fig. 1B; dashed arrow). Immunobloting analysis of purified vesicles verified the presence of high amounts of the mature conventional sCLU isoform of ∼40 kDa at the isolated vesicular material (N = 2, Fig. 1C) confirming thus sCLU exocytosis to vesicles released during RBCs *in vivo* membrane vesiculation.

### Progressive depletion of erythrocytes in sCLU during *ex vivo* cellular aging is due to sCLU selective accumulation in RBCs-derived vesicles

**Figure 2:**
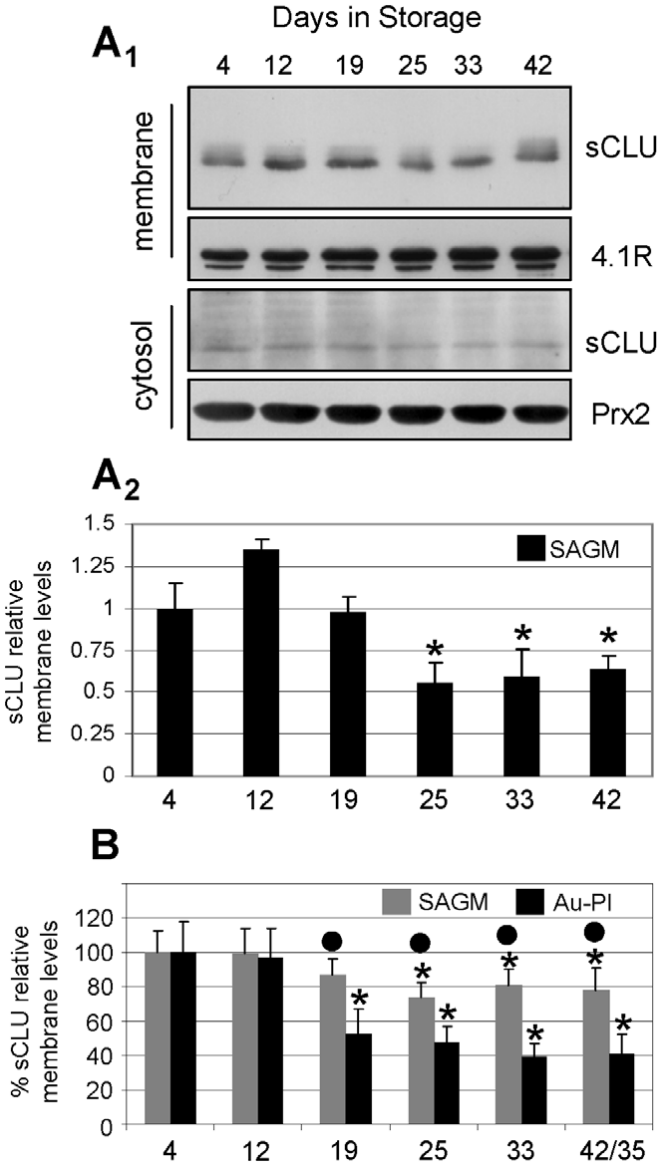
Progressive decrease in the erythrocytes sCLU levels during *ex vivo* cellular senescence. Representative sCLU immunoblot (A_1_) and densitometric analysis (A_2_) of membrane (A_1_, upper panels) and cytosol (A_1_, lower panels) preparations derived from leukoreduced RBCs units (N = 4) stored for the indicated duration in SAGM solution. (B) Comparative densitometric analysis of immunoblots (not shown) of sCLU relative membrane levels in RBCs stored either in SAGM (N = 4; max 42 days of storage) or autologous plasma (Au-Pl) (N = 3; max 35 days of storage). Probing with anti-4.1R and anti-peroxiredoxin-2 (Prx2) was used as a protein loading reference. Shown densitometric data (mean values of at least two independent experiments) indicate relative proportion against a loading reference followed by normalization against the samples stored for a short period (e.g. 4 days); error bars indicate ± standard deviation. Asterisks and dots indicate difference of each day of storage vs. day 4 and SAGM vs. Au-Pl respectively, at significance level of p<0.05.

**Figure 3:**
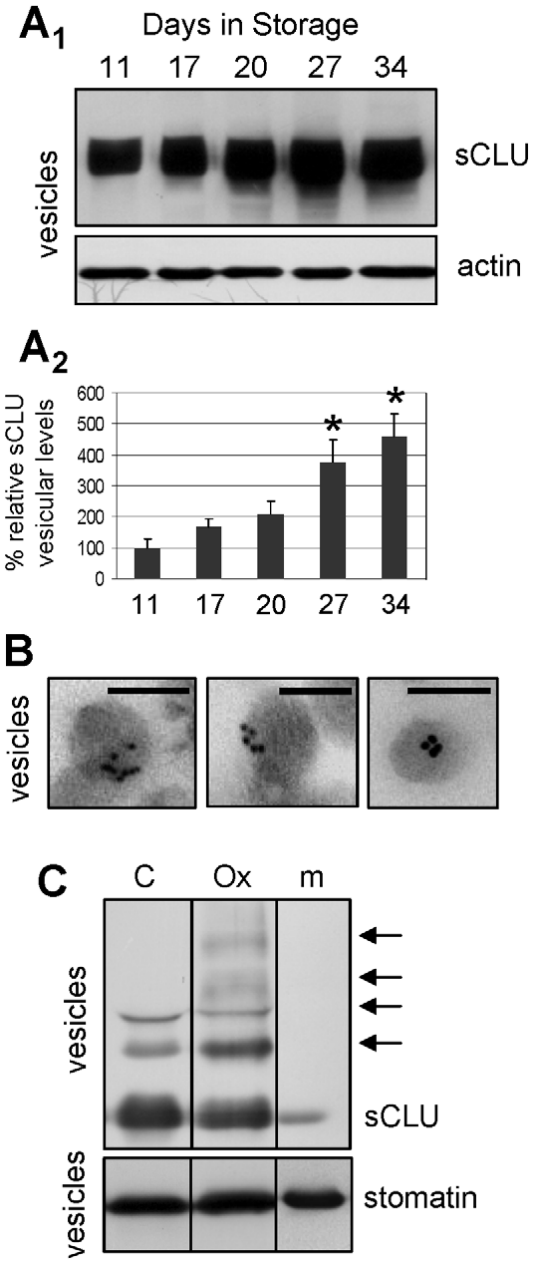
Progressive sCLU accumulation in the RBCs-derived vesicles released during *ex vivo* aging in blood bank storage conditions. Representative sCLU immunoblot (A_1_) and densitometric analysis (A_2_) of vesicle preparations derived from RBCs units stored in autologous plasma (N = 4). (B) TEM immunogold localization of sCLU at the periphery and the cytosol of vesicles derived from RBCs stored in autologous plasma for 35 days (N = 2). (C) *In vitro* analysis of sCLU oligomerization pattern (arrows) in vesicles derived from control (C) or tBHP oxidized (Ox) RBCs; m, denotes an isolated plasma membrane sample. Probing with anti-actin and anti-stomatin were used as protein loading references. Shown densitometric data of sCLU vesicular levels (mean values of at least two different experiments) indicate relative proportion against a loading reference followed by normalization against the controls, namely samples stored for a short period of 11 days; error bars indicate ± standard deviation. Asterisks indicate difference of each day of storage vs. day 11 at significance level of p<0.05. Bars in (B), 100 nm.

To ascertain whether sCLU exocytosis in RBCs-derived vesicles is a continuous and progressive process during the cellular aging, we firstly studied sCLU kinetics during RBCs storage in blood bank conditions. As previously reported, the system of *ex vivo* RBCs storage resembles *in vivo* cellular aging due to occurrence of comparable cellular changes and extensive vesiculation (see introduction). By using immunobloting analysis, sCLU was detected in decreasing amounts in the membrane (Fig 2A_1_, upper panel) and in the cytosol (Fig 2A_1_, lower panel) of leukodepleted RBCs stored in units containing the additive solution SAGM (N = 4). The reduced levels of sCLU during RBCs senescence *ex vivo* were mainly evident after the second half of the storage period (days 25–42, p<0.050 vs. day 4, Figs 2A_1_, 2A_2_), which coincides with the previously reported increase in RBCs vesiculation [9], [15], [16]. Compared to storage in autologous plasma, the addition of mannitol-containing SAGM solution effectively reduces hemolysis, vesiculation, oxidative stress, membrane instability and the senescing rate of RBCs, thus ensuring longer and better storage of RBCs [15], [19]. Consequently, we then examined whether the kinetics of sCLU membrane levels is diversified in RBCs stored in the absence of the beneficial effects of mannitol. We found that, in comparison to cells stored in SAGM, the RBCs stored in autologous plasma (N = 3) were characterized by a higher rate of membrane sCLU loss during the storage period (Fig. 2B). Compared to the first days of storage and starting on day 19, the membrane levels of sCLU in Au-Pl RBCs were lower (p<0.050) as compared to the expression levels in cells stored in SAGM. The reduction in the sCLU membrane levels at the end of the storage period, namely, day 42 for SAGM and day 35 for autologous plasma, was found to be ∼23% vs. ∼58%, respectively. To definitely demonstrate that sCLU is exocytosed during the whole period of *ex vivo* cellular senescence we recorded sCLU content in the exovesicles released into the supernatant of the RBCs units from day 11 to day 34 of storage (N = 4). Immunoblotting analysis showed that the sCLU amount in the released vesicles increased (by up to ∼4.5 fold at the last week of storage as compared to the first days) mainly after the second half of the *ex vivo* erythrocytes lifespan (Fig. 3A); this pattern of sCLU accumulation in RBCs-derived vesicles coincided with the observed reduction of sCLU levels in the stored RBCs (see, Fig. 2). TEM immunogold localization of sCLU in vesicles collected after 35 days of storage in autologous plasma (N = 2) revealed that vesicular sCLU localizes primarily at the periphery but also at the cytosol of the vesicles as well (Fig 3B).

Previous studies have suggested that RBCs *ex vivo* senescence and vesiculation processes are accompanied by a progressive increase in the membrane proteome oxidative defects [15], [20], [21]. As shown in Fig. 3C, exposure of isolated stored RBCs (N = 3, on 27 day of storage) to the oxidant tBHP resulted in increased sCLU presentation and oligomerization in vesicles derived from the oxidized RBCs, implying a role of oxidative stress into sCLU exocytosis to vesicles.

### sCLU binds to Band 3, CD59, Hb and a range of carbonylated-oxidized RBCs membrane proteins

**Figure 4:**
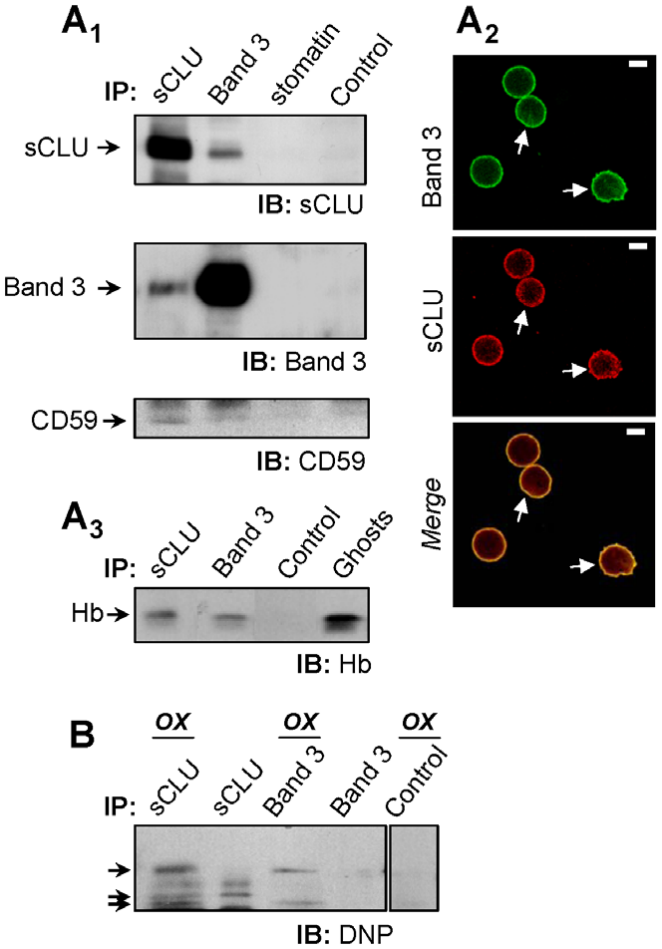
sCLU interactions in RBCs membrane. Purified RBCs membranes from healthy subjects (N = 6) were lysed in NP-40 and lysates were immunoprecipitated (IP) with polyclonal antibodies against sCLU, Band 3, stomatin or normal serum (control). Immunoprecipitates were immunoblotted (IB) under reducing conditions for sCLU (A_1_, upper panel), Band 3 (A_1_, middle panel), CD59 (A_1_, lower panel) and Hb (A_3_); shown IPs are representatives from two independent experiments. (A_2_) CLSM co-immunolocalization of the sCLU and Band 3 proteins at the RBCs plasma membrane. Cells were co-stained with anti-Band 3 monoclonal (green; upper panel) and anti-sCLU polyclonal antibodies (red; lower panel). Captured images were merged to reveal co-distribution sites (yellow; lower panel, arrows). Bars, 3 µm. (B) Anti-dinitrophenylhydrazone (DNP) immunoblotting of sCLU, Band 3, and control (IgGs) immunoprecipitates for the detection of co-immunoprecipitated carbonylated proteins (arrows) in 2,4-dinitrophenylhydrazine-modified (OX) or unmodified protein material.

The previous results outlined a probable role for sCLU in the molecular processes of senescence and vesiculation in RBCs. Considering the chaperone function of sCLU [4] we screened for sCLU binding partners among a group of RBCs proteins that are functionally implicated in those processes. By using co-immunoprecipitation analysis (N = 6, young subjects), we showed that immunoprecipitation of sCLU or Band 3 in membrane preparations resulted in co-immunoprecipitation of Band 3 or sCLU respectively, indicating a physical interaction of Band 3 and sCLU in RBCs plasma membrane (Fig. 4A_1_). Subsequent CLSM immunofluorescence co-immunolocalization studies verified the co-localization of the two proteins at the RBCs plasma membrane (Ν = 3, Fig. 4A_2_). By applying the same analysis we also found that sCLU interacts with CD59 (Fig. 4A_1_, lower panel), a glycosyl phosphatidylinositol-anchored complement regulation protein that localizes at the extracellular side of the RBCs membrane as well as with Hb (Fig. 4A_3_). On the contrary, sCLU does not bind stomatin (Fig. 4A_1_) a protein embedded at the cytosolic side of the RBCs membrane and exocytosed through vesiculation. In accordance to our recent studies showing that sCLU does not distribute in the RBCs cytoskeleton (Antonelou et al., accompanying paper) we found no sCLU interaction with several cytoskeleton constituent proteins examined (data not shown). As it has been proposed that sCLU binds and stabilizes stressed proteins in a folding-competent state [4] we asked whether sCLU also binds carbonylated proteins at the RBCs plasma membrane. By applying limited 2,4-dinitrophenylhydrazine (DNPH) modification of the sCLU- or Band 3-immunoprecipitated material followed by dinitrophenylhydrazone (DNP) immunoblotting, we detected a range of carbonylated-oxidized bands in association to both sCLU and Band 3 proteins (Fig. 4B).

## Discussion

sCLU is a primarily secreted protein sorted for secretion through the classic endoplasmic reticulum- and Golgi-associated secretory pathway [5], [17], [22], [23]. Our currently reported findings provide evidence that sCLU is also a “secreted” component of mature RBCs through the membrane exovesiculation process. Furthermore, in common to the protective role that sCLU exhibits in other mammalian tissues, wherein it functions to alleviate cells from the deleterious effects of increased oxidative stress [1], [2], the pattern of sCLU protein interactions and exovesiculation, delineates its active implication in the disposal of oxidized/defected molecules from maturing RBCs.

We have reported a significant loss of sCLU membrane levels in senescent or stressed RBCs *in vivo*, both in physiological conditions as well as in hemolytic disease (Antonelou et al., accompanying paper). The blood bank storage has been widely used as an alternative model system of *in vivo* aging [9]. In fact, some aspects of the erythrocyte aging may be more pronounced and therefore easier studied in units of stored cells than in freshly obtained cells because of the absence of an immunological removal system [24]. Our studies on sCLU of RBCs stored in blood bank conditions verified that prospect and indicate that the decrease in the sCLU levels refers to both the membrane and the cytosolic sCLU. Moreover, the gradual loss of sCLU from RBCs is a progressive and continuous process, throughout the maturation stages, related to endogenous RBCs-specific molecular processes of aging, redox imbalance and vesiculation rate.

According to both *in vivo* (Antonelou et al., accompanying paper) and currently reported *ex vivo* data, the decrease in the RBCs sCLU levels takes place via selective sorting and exocytosis of sCLU through vesiculation and is an aging- and oxidative stress-related mechanism. Supportive information resulted by both the analysis of the RBCs-derived vesicles and the co-immunoprecipitation experiments. Indeed, by *in situ* approaches we demonstrated the constant localization of sCLU at both the membrane and the cytosolic compartment of the RBCs-derived vesicles from the very first day of storage and furthermore the progressive accumulation of the protein into the vesicles towards the completion of the storage period. The monitoring of sCLU accumulation in vesicles coincides with the progressive depletion seen in the stored RBCs and the previously documented progressive increase in vesiculation and proteome carbonylation defects [16], [20], [21]. In addition, the co-immunoprecipitation analyses of sCLU not only verified the previously reported data on patients with hemolytic anemia (Antonelou et al., accompanying paper), which indicated that membrane association of the protein in RBCs may depend on factors other than IgGs, but also demonstrated that all of the observed physical interactions of sCLU are known components of the RBCs-derived vesicles, namely Band 3, CD59, Hb and carbonylated proteins.

A central role for sCLU in the regulatory networks of aging RBCs has been recently predicted following *in silico* establishment of the interactome models of RBCs proteins [25]. Specifically, it was predicted that sCLU interacts with several proteins in the core of the network, including chaperones, caspases and CD59 in the “Cell Death” sub-network [25]. CD59 is a glycosyl phosphatidylinositol-anchored and highly mobile complement regulation protein of the RBCs surface and a component of RBCs-derived vesicles [8]. Apart from its role in membrane vesiculation [26], CD59 has been also implicated in the inhibition of the lytic membrane attack complex assembly [27]. Although the functional consequences of the sCLU binding to CD59 are not clear, the currently observed sCLU-CD59 physical interaction may relates to the recently reported sCLU-mediated inhibition of the membrane attack complex and rabbit erythrocytes hemolysis [28]. On the other hand, Band 3 plays a key role in regulating RBCs metabolism, membrane structure and ion exchange while its modifications are tightly associated with RBCs aging, vesiculation, erythrophagocytosis and cell death [9], [11], [24]. Specifically, the recognition and removal of senescent RBCs is triggered by binding of autologous IgGs to a neo-antigen that originates from Band 3 protein modifications. The Hb present in the sCLU-immunoprepicipated material represents either independent binding of intracellular sCLU to the membrane or cytosolic Hb or most probably the co-immunoprecipitated Band 3-bound Hb molecules. Overall, the binding profile of sCLU in RBCs membrane implies a specific role for sCLU in the exovesiculation process and the management of stress imposed by either endogenous or exogenous factors in RBCs. The comparative monitoring of sCLU membrane levels in two storage systems (Au-Pl and SAGM) that differ between each other in vesiculation degree, membrane proteome oxidation defects and cellular aging progression [15], [19], further supported the impact of all of these parameters on the sCLU loss in stressed, maturing or senescent RBCs.

Moreover, our co-immunoprecipitation data documented the physical association of sCLU with a range of carbonylated proteins in the RBCs membrane. Considering that, sCLU functions as a chaperone involved in the quality control of protein folding [29] and in the clearance of cellular debris by non-professional phagocytes [3] we propose that it also contributes to the scavenging of oxidized or aggregated molecules that are selectively removed from senescent or stressed RBCs via vesiculation. By assuming that, sCLU represents not only a molecular biomarker of cellular senescence and oxidative stress but also a pro-survival factor that contributes to the transient inhibition or delay of the premature removal of otherwise functional RBCs from the circulation; this proposed function of sCLU is further supported by the recently predicted role of the protein in the RBCs death regulatory pathways [25]. Such a lifetime-extension mechanism might be especially beneficial to individuals with hemolytic diseases and to those who receive transfusion of long-term stored RBCs.

**Figure 5:**
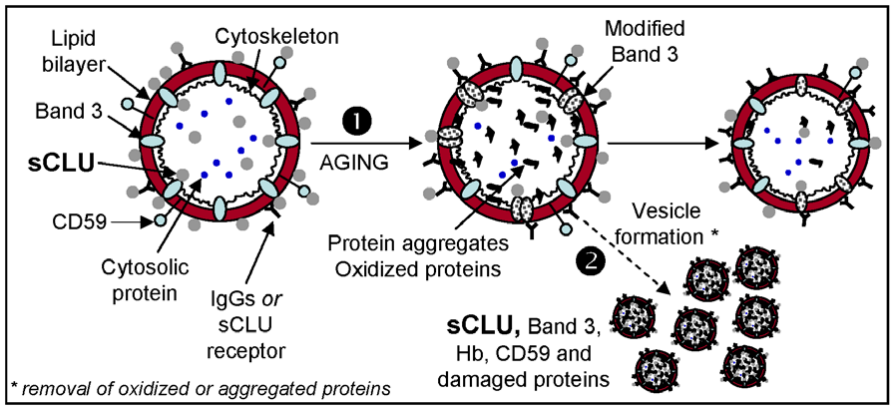
Proposed model of sCLU involvement in RBCs plasma membrane integrity, cellular senescence and vesiculation. Erythrocytic sCLU localizes at both sides of the plasma membrane in association with non-cytoskeletal areas, as well as in the cytosol (see also, Antonelou et al., accompanying paper). At the intracellular side of the RBCs membrane sCLU may bind Band 3, Hb and/or other cytoskeleton-free membrane portions. On the other hand, the sCLU that localizes at the extracellular side of the RBCs membrane can attach to membrane by binding to Band 3, CD59, plasma membrane IgGs or to an currently unknown sCLU-specific receptor. Physiological *in vivo* or *ex vivo* RBCs senescence (1) is associated with cytosol, cytoskeleton and membrane structural alterations, including Band 3 modifications, increased membrane binding of IgGs, proteolysis, protein aggregation and increased oxidation defects. Vesiculation (2), a self-protective mechanism of mammalian erythrocytes, removes oxidized proteins and aggregates from both plasma membrane and cytosol thereby postponing the untimely elimination of otherwise healthy erythrocytes. This process takes place through the entire *in vivo* or *ex vivo* lifespan of RBCs and is functionally connected to the release of sCLU-, Band 3-, CD59-, Hb- and IgGs-containing vesicles. We propose that vesicular sCLU by following its membrane linkers (e.g. Band 3) or other unknown cytosolic interacting proteins assists via its chaperone function in the disposal of non-functional or death signalling effective material from RBCs.

Taken together, our results (summarized in Fig. 5) provide novel evidence for an emerging role of sCLU in the physiology of mature human erythrocytes and the molecular processes involved in RBCs aging and vesiculation. The maturation of RBCs is associated with a progressive loss of sCLU. We propose that during RBCs aging sCLU, most probably through its interaction with Band 3, CD59, Hb and carbonylated membrane molecules, is actively involved in the disposal of oxidized and/or defected material through vesiculation. As clinical practice demands for biomarkers of *in vivo* blood aging as well as of *in vitro* labile blood products storage lesions evaluation and post-transfusion effects, our data might be of high interest for the transfusion medicine community. Indeed, sCLU content in stored RBCs would appear to be a useful tool in assessing the impact of changes in the blood banking manufacturing process. Work in progress in our lab aims to investigate these issues in a well-controlled setting, by using a murine RBCs storage and transfusion model.
